# Size Control of Carbon Xerogel Spheres as Key Factor Governing the H_2_O_2_ Selectivity in Metal-Free Bifunctional Electro-Fenton Catalysts for Tetracycline Degradation

**DOI:** 10.3390/gels10050306

**Published:** 2024-05-01

**Authors:** Edgar Fajardo-Puerto, Nerea López-García, Abdelhakim Elmouwahidi, Esther Bailón-García, Francisco Carrasco-Marín, Lilian D. Ramírez-Valencia, Agustín F. Pérez-Cadenas

**Affiliations:** Materiales Polifuncionales Basados en Carbono, Departamento de Química Inorgánica—Unidad de Excelencia de Química Aplicada a Biomedicina y Medioambiente—Universidad de Granada (UEQ-UGR), 18071 Granada, Spain; nerealopezg7@correo.ugr.es (N.L.-G.); aelmouwahidi@ugr.es (A.E.); fmarin@ugr.es (F.C.-M.); liliandr@correo.ugr.es (L.D.R.-V.); afperez@ugr.es (A.F.P.-C.)

**Keywords:** wastewater, ORR, electro-Fenton, carbon gels, xerogels

## Abstract

Carbon xerogel spheres co-doped with nitrogen and eco-graphene were synthesized using a typical solvothermal method. The results indicate that the incorporation of eco-graphene enhances the electrochemical properties, such as the current density (J_K_) and the selectivity for the four transferred electrons (n). Additionally, nitrogen doping has a significant effect on the degradation efficiency, varying with the size of the carbon xerogel spheres, which could be attributed to the type of nitrogenous group doped in the carbon material. The degradation efficiency improved in the nanometric spheres (48.3% to 61.6%) but decreased in the micrometric-scale spheres (58.6% to 53.4%). This effect was attributed to the N-functional groups present in each sample, with N-CNS-5 exhibiting a higher percentage of graphitic nitrogen (35.7%) compared to N-CMS-5 (15.3%). These findings highlight the critical role of sphere size in determining the type of N-functional groups present in the sample. leading to enhanced degradation of pollutants as a result of the electro-Fenton process.

## 1. Introduction

Low rainfall and high temperatures are leading to increasingly extreme drought conditions. In this context of water scarcity, wastewater reuse is essential. However, the presence of emerging pollutants in water makes its reuse difficult, as such pollutants represent an environmental and human health risk [1]. Antibiotics are being detected in the wastewater in increasing amounts due to the rapid development of urbanization, which generates a more serious problem in the form of antibiotic resistance genes [2,3]. Conventional treatment plants cannot effectively remove these pollutants, so there is an urgent need to develop effective technologies to address this problem [4,5]. The advanced oxidation processes (AOPs) have shown excellent results in the degradation of antibiotics, thanks to the generation of hydroxyl radicals (OH). Specifically, the electro-Fenton (EF) process has been studied due to its advantages associated principally with the risks, transport, and storage of peroxide hydrogen (H_2_O_2_), since the H_2_O_2_ is produced directly in the cathode of EF [6] in mild conditions and does not involve other hazardous materials [7]. The electro-generation of H_2_O_2_ in EF is based on the oxygen reaction reduction (ORR) via two electrons (2e^−^) pathway (Equation (1)).

O_2_ + 2H^+^ + 2e^−^ → H_2_O_2_ (1)

However, the main problems associated with the EF are the slow regeneration of Fe(II) (Equation (3)), which is responsible for the H_2_O_2_ transformation into ·OH (Equation (2)), and the competitive route of ORR four electrons (4e^−^) (Equation (4)) which decrease the H_2_O_2_ production, and therefore the overall efficiency of the process [8,9,10].

Fe(II) + H_2_O_2_ → Fe(III) + ·OH+ OH^−^ (2)

Fe(III) + e^−^ → Fe(II) (3)

O_2_ + 4H^+^ + 4e^−^ → 2H_2_O (4)

Carbon materials are promising candidates as electrodes for 2e^−^ ORR due to carbon’s good properties such as high electrical conductivity, electrochemical stability, low cost, non-toxicity, high overpotential for H_2_ evolution, and low decomposition of H_2_O_2_ [11,12]. Some carbon materials used in the H_2_O_2_ electro-generation with promising results included graphite modified with polypyrrole/multiwalled carbon nanotube (MWCNT) [13], inks based on different carbon materials (activated carbon, carbon graphite and carbon black) [14], vulcan XC-72 carbon with niobium oxide (Nb_2_O_5_) [15] and biochar’s [16,17], among others. An alternative that is usually used to improve the H_2_O_2_ production is nitrogen (N) doping/functionalization. Y. Zhang et al. [18] synthesized a hierarchical porous O, N co-doped porous carbon nanosheet (ONPC) for the selective reduction of O_2_ to H_2_O_2_, demonstrating that the pyrrolic-N and C=O motifs enhance the H_2_O_2_ generation. F. Wu et al. [19] also synthesized an N, O co-doped graphite nanosheet, corroborating that the different oxygen-containing functional groups and N species affect the H_2_O_2_ generation. In this case, the combination of epoxy and graphitic N was identified as the most favorable configuration with the lowest theoretical overpotential for H_2_O_2_ generation. In turn, X. Wang et al. [20] used density functional theory (DFT) calculations to analyze the effect of O, N co-doping of carbon nanosheets on the H_2_O_2_ selectivity. They demonstrated that the binding strength of *OOH was optimized by the co-doping of oxygen and nitrogen at certain content, and that the O/N-C_COOH site exhibits a lower theoretical overpotential for H_2_O_2_ formation than O-C_COOH site. Thus, besides the nitrogen doping level, the nitrogen speciation is also crucial for the selective reduction of oxygen to hydrogen peroxide (ORR to H_2_O_2_). Various nitrogen configurations can exist in nitrogen-doped carbon materials, such as N-pyridinic, N-pyrrolic, N-graphitic, N-quaternary, and N-oxide [21]. Y. Yang et al. [22] investigated the efficiency of a nitrogen-doped carbon electrocatalyst in selectively producing H_2_O_2_ from O_2_, suggesting that disordered carbon defects and pyrrolic-N structures are pivotal in enhancing H_2_O_2_ generation. S. Han et al. [23] also evidenced that the nature of the nitrogen groups is key to controlling the H_2_O_2_ selectivity. They showed that Pyridinic-N and graphitic-N enhanced the 2e^−^ ORR selectivity of the cathode and promoted ·OH generation at acidic pH. Y. Zhu et al. [24] synthesized N-doped carbons by pyrolysis of three N-precursors (2,6-diaminopurine, 2,4,6-tripyridin-2-yl-1,3,5-triazine and 1h-1,2,4-triazole-3,5-diamine). All N-doped carbons presented enhanced H_2_O_2_ production in relation to graphite, since N doping provides active sites for oxygen reduction, improving ORR activity. However, the types and proportions of N-functional groups are different in the three materials. All samples contained a high amount of a high proportion of pyrrolic N content, which favors the H_2_O_2_ accumulation. However, the sample prepared from 1h-1,2,4-triazole-3,5-diamine showed the highest H_2_O_2_ accumulation among the three nitrogen-doped cathodes, which was attributed to the high electroactive surface area and pyrrolic N (60.45%) incorporation. Y. Sun et al. [25] studied a series of nitrogen-doped porous carbon materials for ORR to H_2_O_2_, indicating that pyridinic-N plays a crucial mechanistic role in acidic conditions, whereas graphitic-N acts as the active site in neutral and alkaline environments. J. Zhang et al. [26] identified graphitic-N as the active site for the 2e^−^ ORR to H_2_O_2_ on nitrogen-doped carbons. Interestingly, these same nitrogen species have been proposed as active sites for the 4e^−^ ORR to H_2_O. Consequently, the significance of nitrogen speciation in promoting ORR remains contentious and subject to debate. It is probable that a combination of carbon defects and specific nitrogen sites facilitates the 2e^−^ pathway for ORR to H_2_O_2_. However, more extended observations in the literature have indicated that N-pyridinic tends to enhance the four-electron oxygen reduction reaction (ORR) by facilitating electron donation, whereas N-pyrrolic aids in accelerating the two-electron ORR [27,28]. Additionally, graphitic-N has also been identified as the superior active site for the 2e^−^ ORR to H_2_O_2_. Regarding oxidized N, simulated calculations from other research suggest that the free energy of oxygen adsorption in the ORR process on oxidized nitrogen is notably higher compared to that of other nitrogen species, indicating its unfavorable contribution to H_2_O_2_ generation [29].

Although H_2_O_2_ generation is improving, the EF process still necessitates the utilization of two catalysts: one tailored for oxygen reduction to H_2_O_2_ and another Fenton-type catalyst for converting H_2_O_2_ to hydroxyl radicals. In recent years, there have been numerous endeavors to create materials that possess dual functionality for electroreduction of oxygen to H_2_O_2_ and Fenton reactions. However, crafting heterogeneous EF catalysts with high selectivity and activity towards ORR through the two-electron pathway remains challenging. This is because transition metals, which are primarily responsible for Fenton reactions, typically catalyze oxygen reduction via the 4e^−^ pathway, which does not yield H_2_O_2_. To overcome these limitations, researchers worldwide have recently started to study the use of carbonaceous materials as possible bifunctional catalysts, which are capable of directly generating OH• without the need for transition metal or Fenton-type catalysts.

As shown above, carbonaceous materials are optimal catalysts for ORR 2 e^−^. However, it has recently been shown that well-developed mesoporosity can be more beneficial for ORR, as unlike nano- or micropores, which can easily be blocked, mesopores allow adequate transfer of reactants, products and/or electrons, which can lead to the reduction of H_2_O_2_ with an additional electron (Equation (5)) [30].

H_2_O_2_ + e^−^ → ·OH+ OH^−^ (5)

Tan et al. [31] demonstrated that nitrogen-doped porous carbon (NPC) can activate H_2_O_2_ to ·OH, which was attributed to the enhanced hydrogen peroxide adsorption on the N-graphitic sites. They [32] also showed the bifunctionality of ordered mesoporous carbon, which proved to be active for ·OH generation from ORR; the catalytic activity was associated with the nano-confinement in the mesoporous structure and C-O-C groups. Carbonaceous materials doped with transition metals (Fe or Mn) have also been proposed as catalysts for the in situ generation of ·OH via a three-electron ORR reaction (Equation (6)) [33,34].

O_2_ + 3e^−^ → OH•+ H_2_O (6)

In this work, free-metals bifunctional electrocatalysts based on carbon spheres doped with N functional groups were synthesized and their behavior as electrodes in the ORR 3e^−^ was analyzed with regard to the degradation of tetracycline, which was used as an antibiotic reference. The effect of the carbon microsphere size on the stabilization of different N functional groups, and consequently, the electro-Fenton activity, as well as the improvement of conductivity of samples by adding eco-graphene, were deeply analyzed. The control of the selectivity to three- or two-electrons pathways, which was achieved by changing the operational voltage, was also evaluated.

## 2. Results and Discussion

### 2.1. Morphological and Textural Characterization

#### 2.1.1. Morphology

**Figure 1:**
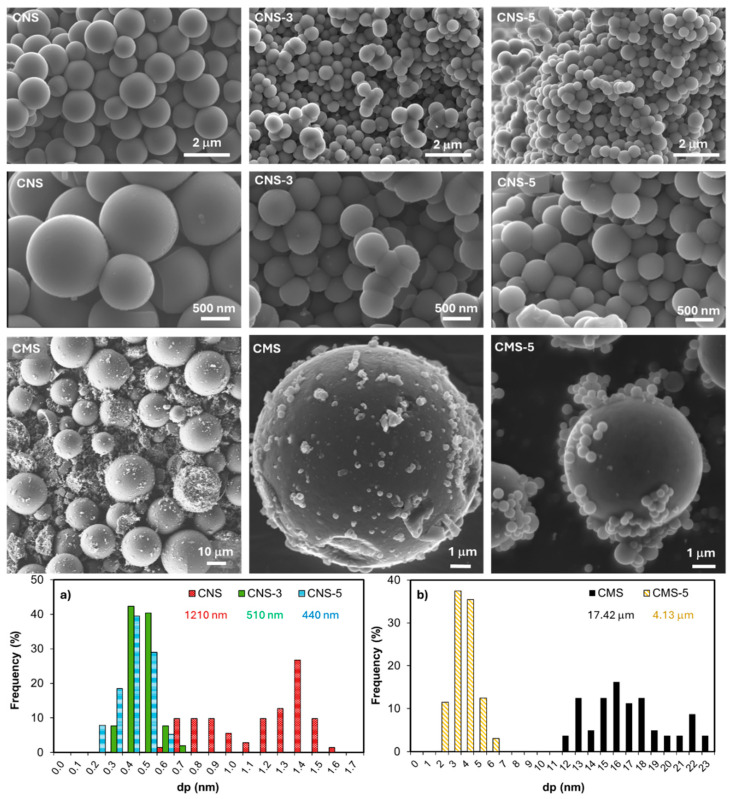
SEM images of carbon nano- (CNS) and macrospheres (CMS) without eco-graphene and with 3% (CNS-3%) and 5% (CNS-5 and CMS-5) of eco-graphene and particles size distribution of nano (**a**) and micro-sized spheres (**b**) obtained from analysis of SEM images.

SEM images of bare carbon nano- and microspheres (CNS and CMS, respectively) and eco-graphene (EG)-doped carbon spheres (CNS-3, CNS-5 and CMS-5) are collected in Figure 1. Isolated spheres smaller than 1.6 μm were obtained via the hydrothermal method (CNS), whereas spheres of up to 24 μm were obtained via the inverse emulsion method [35] (CMS). Notably, a cleaner and more perfect surface was observed in CNS in comparison to CMS, with which a more rugous and imperfect surface was obtained. This result could be associated with the oxygen concentration in each sample. It has been reported that hydrophilicity and roughness are inversely proportional to the oxygen amount [36], which can be derived from the synthesis method, where according to the literature, the solvothermal method, due to high temperature and pressure, can incorporate oxygen into the final material [37]. In both seria, CNS and CMS, doping with EG affected the sphere size distribution, causing the size to decrease with a concurrent increase in the EG doping. The size of CNS decreased from a mean size of 1.21 μm to 510 and 440 nm with the addition of 3 and 5 wt.% of EG, respectively; the size of CMS also decreased from a mean size of 17.42 μm to 4.13 μm when 5 wt.% of EG was added. The EG could interfere in the sol–gel polymerization, creating nucleation centers which favor the creation of more spheres of smaller size instead of a sphere’s growth. In any case, EG seems not to affect the shape or rugosity of the spheres.

**Figure 2:**
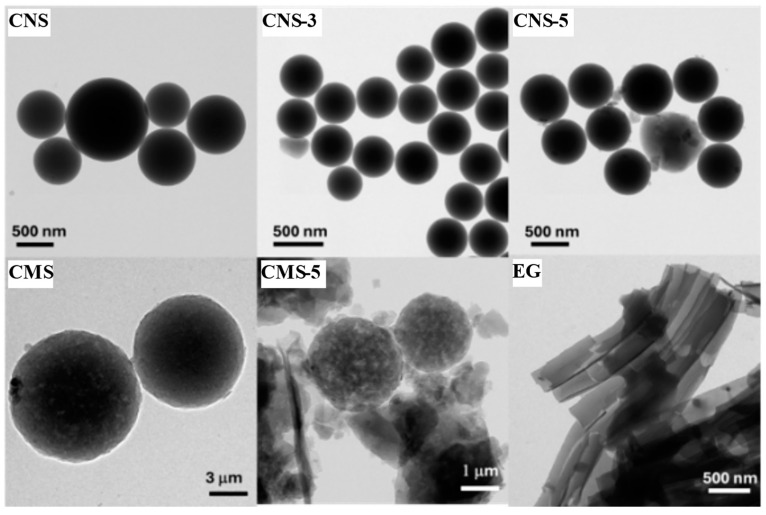
TEM images of eco-graphene (EG) and carbon nano- (CNS) and macrospheres (CMS) without eco-graphene and with 3% (CNS-3%) and 5% (CNS-5 and CMS-5) eco-graphene.

TEM was used to try to observe the EG distribution in the samples. TEM images are collected in Figure 2. The same findings can be derived from the analysis of TEM images. Once again, larger particles are evident in the non-EG doped samples (CNS and CMS), whereas spheres that are smaller in size are observed in samples doped with eco-graphene. However, significant differences could be observed between micro and nanospheres. Nanospheres seem to be more dense and less porous, with more uniform surfaces than microspheres, on which surface rugosity and internal voids can be clearly identified. In turn, the synthesized eco-graphene (EG) comprises smooth and seemingly defect-free nanosheets. It is noteworthy that some eco-graphene sheets are uniformly distributed in close proximity around the spheres in all eco-graphene-doped samples, which could allow a better distribution of loads or electronic transfer, which would be beneficial for the ORR.

#### 2.1.2. Textural Characterization

**Figure 3:**
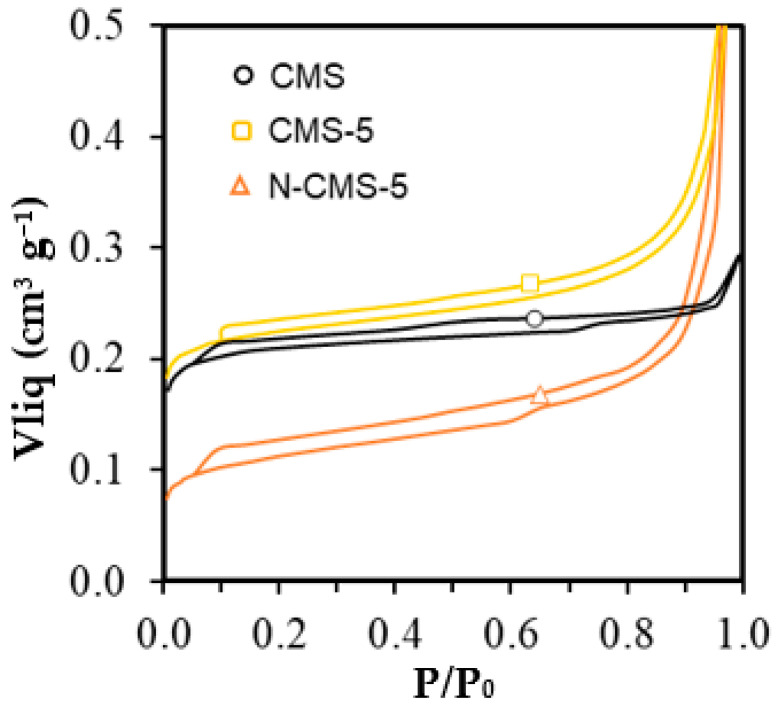
N_2_-adsorption/desorption isotherms CMS series.

The porous texture of samples was analyzed by N_2_ and CO_2_ adsorption at −196 and 0 °C, respectively. N_2_ isotherms are depicted in Figure 3 and results from the data analysis are included in Table 1. A type I isotherm is obtained, denoting the presence of micropores in all samples. In samples doped with EG (both micro and nanospheres), there is an increase in N_2_ adsorption at intermediate relative pressures, which indicates the creation of some mesoporosity in the samples by the addition of EG (V_meso_, Table 1). Moreover, the N-doping results in a reduction in microporosity due to the blockage of wider micropores by the fixed N-functional groups. It is important to highlight that despite both CMS and CNS presenting a similar surface area and pore volume, carbon nanospheres are mainly ultramicroporous with micropores of around 0.51 nm, whereas carbon microspheres present wider micropores of a mean size of 1.19 nm.

#### 2.1.3. Raman Characterization

**Figure 4:**
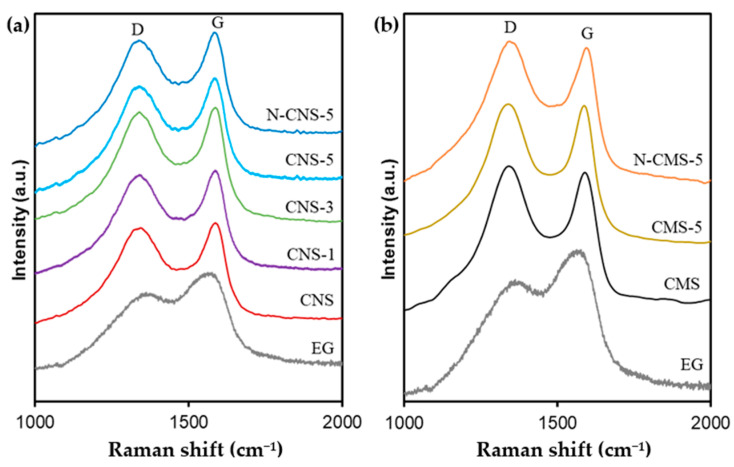
Raman spectra of (**a**) CNS and (**b**) CMS series.

The degree of graphitization of all samples was analyzed by Raman spectroscopy and the results are collected in Table 1 and Figure 4. Two bands at around 1345 and 1587 cm^−1^ were identified: these are referred to as defect (D) and graphitization (G) bands, respectively. The G peak arises from the in-plane stretching motion among sp2 carbon atoms, whereas the D band is attributed to structural defects, edge effects, and unpaired sp2 carbon bonds, disrupting the symmetry [38]. The variation in the position, width, and intensity of the Raman bands is used to determine the structural order of the material. The intensity ratio I_D_/I_G_ is indicative of carbon structural ordering, with a significantly lower ratio correlating to a higher degree of graphitization. According to the Raman spectra (Figure 4), the change in the band position of D and G peaks and the intensity ratio (I_D_/I_G_) in the pure eco-graphene and EG-doped carbon spheres clearly confirm the different degrees of graphitization. Note that the G peak is much intense than the D peak for EG, showing a low I_D_/I_G_ ratio (0.78), which denotes the high graphitization degree of eco-graphene. The I_G_ is also significantly higher than I_D_ for carbon nanospheres (CNS) in contrast to CMS samples where I_D_ is higher than I_G_, denoting a higher defects concentration in carbon microspheres regarding carbon nanospheres. The addition of EG to both carbon spheres (nano and micro) decreases the I_D_/I_G_ ratio, thus increasing the degree of graphitization of carbon spheres to a greater extent at a higher EG concentration. The G band position also shifts to a higher wavenumber with the amorphization degree of samples [38]; thus, the G band shifts from 1563 cm^−1^ in EG to 1588 and 1590 cm^−1^ in CNS and CMS, respectively. This shift is lower at higher EG contents, corroborating the improvement of graphitization of samples with the EG content. It is also important to highlight that the N-doping does not highly affect the graphitization degree of samples, as has been mentioned in the literature [39,40].

### 2.2. Elemental Analysis and XPS Characterization

#### 2.2.1. Elemental Analysis

The elemental composition of samples was analyzed by elemental analysis to identify the amount of nitrogen within the samples after the N-doping with melamine, and the results are shown in Table 2. It is observed that the increase in eco-graphene in both nano and microspheres series increases the nitrogen content. The presence of nitrogen can be attributed to the process of synthesizing eco-graphene. During the hydrothermal process at 270 °C, CTAB molecules decompose, releasing nitrogen and hydrogen gases. These gases interact with the graphitic structure, resulting in nitrogen doping and a reduction in the oxidized structure, respectively. After treatment with melamine, an increase in the N content is observed in all samples. However, the nitrogen content depends on the size of the carbon spheres. The N content of CNS is around 3%, whereas the N fixed in CMS is almost twice that amount. Gong et al. [41] illustrated that imperfections in carbon structures, such as defects, edges, and functionalized carbon atoms, are energetically favorable for the incorporation of nitrogen atoms compared to the basal plane on the surface of carbon materials. Thus, the higher functionalization degree in carbon microspheres could be related to the greater amount of defective and porous surface accessible to the N precursor salt of carbon microspheres, which favors the melamine–surface interaction and consequently, N doping.

#### 2.2.2. XPS Characterization

The surface chemistry of more representative samples was analyzed by XPS. Six peaks are required to fit the C_1s_ region at 284.6 eV, 285.3 eV, 286.6 eV, 288.0 eV, 289.9 eV and 291.7 eV (π-π*) attributed to C=C, C-C, C-O, C=O, COO- and π-π* transition in the aromatic systems, respectively (Figure 4a). Note that the peaks’ positions and peaks’ contributions are quite similar in all samples, indicating that the carbon surface chemistry of samples was not modified after the eco-graphene and N-doping. In turn, the O_1s_ region (Figure 4b) is deconvolved in three peaks assigned to quinone functional groups at 530.8 eV, C=O at 532.3 eV and C-O at 533.5 eV [38]. The presence of quinone functional groups in N- and EG-doped samples is justified by the fixation of these groups after the thermal treatment or by the presence of these functional groups in the added EG. Note that in pure EG (Table 3), quinone functional groups represent 30.8% of O_1s_ spectra. However, it is important to highlight that the quinone contribution is not detected in CMS samples in which nitrogen is not detected by elemental analysis, whereas CNS presents an 8.1% contribution and nitrogen is detected by elemental analysis and XPS, although nitrogen or eco-graphene doping is not performed. This behavior can be explained based on the different synthesis methods used in CMS and CNS samples. In CMS sample, any nitrogen containing reactant is used, whereas urea is used in the hydrothermal synthesis of carbon nanospheres (CNS) which could be decomposed and fixed in the carbon matrix under the pressurized hydrothermal method. The quinone contribution and N content increases, as expected after the nitrogen doping. Note that although the total N content analyzed by elemental analysis in N-doped CMS (6.08 wt.%) is twice that observed for N-doped CNS (2.41 wt.%), the nitrogen content in N-CMS-5 (5.1 wt.%) is lower than in N-CNS-5 (9.1 wt.%). This difference in both techniques is explained based on the N-functional groups’ distribution and carbon spheres’ porosity. Both carbon micro- and nanospheres are microporous materials with similar surface areas. However, wide micropores with a size of 1.19 nm with the presence of some mesoporosity is observed in microspheres whereas mesoporosity is not detected in nanospheres in which ultramicropores (0.51 nm) are present. The wide micropores allow the melamine solution to be accessed at all levels of porosity and thus, N-functional groups are distributed throughout the microspheres with varying porosity, whereas this melamine aqueous solution cannot enter ultramicroporous surfaces, and thus avoids the functionalization of the internal nanospheres’ surface. Since the XPS is a surface technique, only a few nm of the surface is analyzed. The N_XPS_ content (5.1 wt.%) of CNS is much higher than the total N detected by EA (2.41 wt.%) because N-groups are mainly localized on the external surface. In the case of CMS, N_XPS_ (5.1 wt.%) is similar to NEA (6.1 wt.%), since the N-functional groups are distributed homogeneously. 

**Figure 5:**
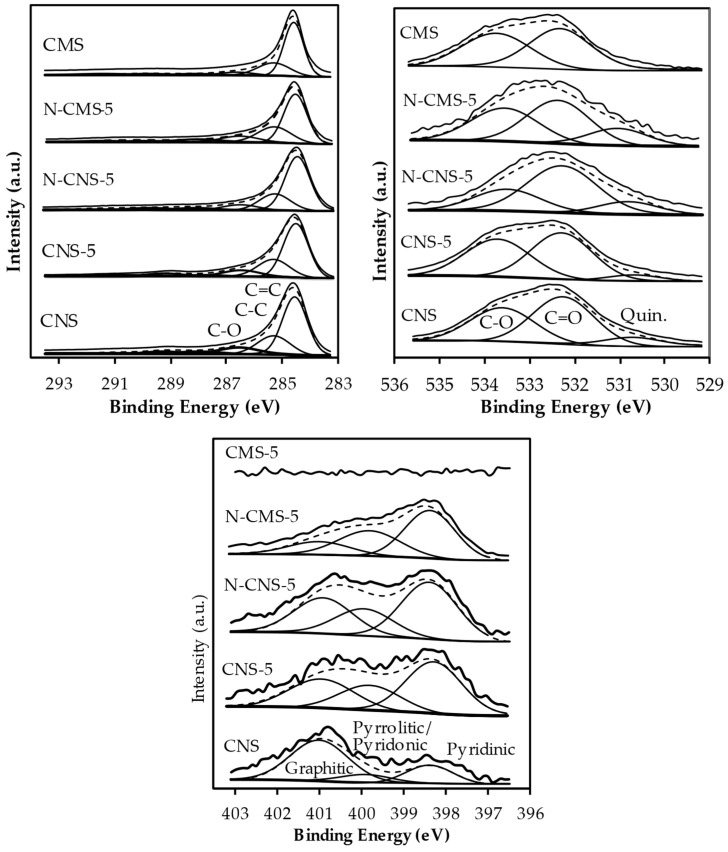
XPS patterns.

The N-containing functional groups of samples are analyzed by deconvolution of the N_1s_ region (Figure 5c). Three peaks are detected at 398.4 eV (pyridinic-N), 399.9 eV (pyrrolitic/pyridonic-N) and 401.0 eV (graphitic-N). Note that the distribution of N-containing functional groups is very different depending on the carbon size (Figure 5c and Table 3). In N-doped carbon microspheres (N-CMS-5), pyridinic-N and pyrrolitic/pyridonic-N are predominant, whereas quaternary-N only represents 15.3%. However, quaternary-N increases significantly in N-doped carbon nanospheres (30.6%) at the expense of a decrease in pyridinic N. Since the nitrogen doping method is the same in both carbon nano- and microspheres, the size of the carbon spheres and consequently, the surface texture and defects seem to be crucial for the selective introduction of graphitic-N. The nature of the N-functional groups determines the ORR selectivity, so the different groups’ distributions in CMS and CNS samples determine their electro-Fenton behavior.

### 2.3. Electrochemical Characterization

#### 2.3.1. Voltammetries

**Figure 6:**
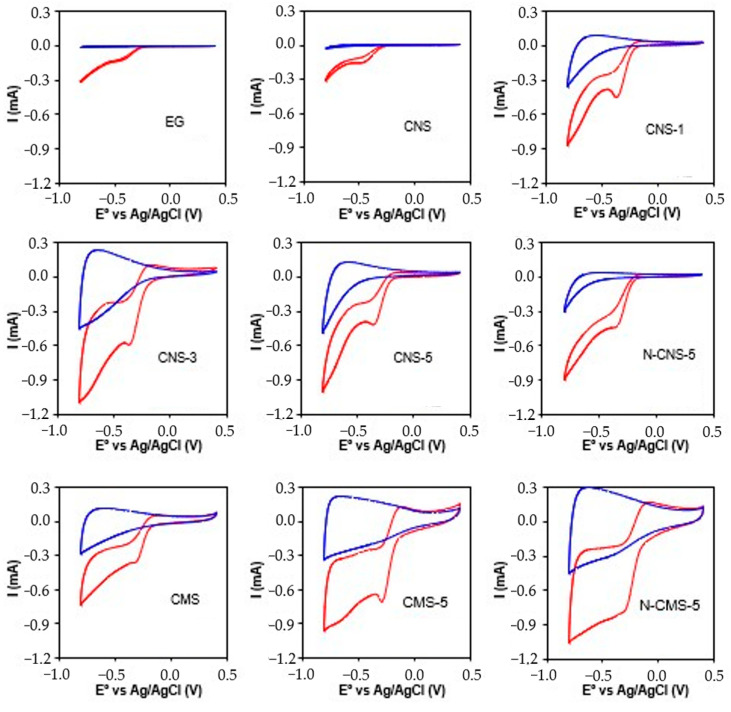
Cyclic voltammograms at 50 mV s^−1^ and 1000 rpm under N_2_ flow (blue line) and O_2_ (red line).

Prior to analyzing the behavior of samples as electrodes for the electro-Fenton degradation of drugs in wastewater, their catalytic performance in the generation of H_2_O_2_ and/or ·OH radicals need to be evaluated. To this end, the oxygen reduction reaction was analyzed using a rotating ring-disk electrode (RRDE). First, cyclic voltammograms were performed under a constant flow of nitrogen and oxygen and the results are shown in Figure 6. At first glance, an increase in the current intensity near −0.2 V vs. Ag/AgCl is observed when the electrolyte is saturated with O_2_ in comparison with N_2_, suggesting that all samples are active in the oxygen reduction reaction (ORR). The varying electrochemical responses of samples under N_2_ bubbling conditions (Figure 6, blue line) may be attributed to differences in conductivity, graphitization degree, textural properties, and surface chemistry [42]. The area enclosed in the cyclic voltammograms (CVs), representing the capacitance, is clearly affected by the EG doping and the size of the spheres. EG presents a very low capacitance due to the reduced surface area on which the electrical double layer could form. Although CMS and CNS present similar textural properties (S_BET_ and W_0_ (N_2_), Table 1) and surface chemistry (elemental composition, Table 2), the capacitance of CMS is much higher than that of CNS. It is well established that the optimal specific capacitance is achieved when pores fall within the range of 0.7−1 nm. Pores smaller than 0.5 nm are too narrow for effective electrolyte diffusion and the formation of double layers [43,44]. Conversely, while mesopores (2−50 nm) may not contribute as significantly as micropores to the formation of double layers, they can enhance the formation of electric double-layer capacitors (EDLC) at high charge rates by facilitating electrolyte diffusion through the carbon network to the active sites within micropores. The pores size in CMS is around 1 nm with the presence of some mesoporosity, whereas ultramicropores of around 0.5 nm without mesoporosity are observed in CNS. This explanation elucidates the distinct capacitance behavior observed in CMS and CNS samples. According to previous studies, it is understood that microporosity can enhance the current diffusion into the pores, thereby increasing capacitance (as evidenced by a higher area of cyclic voltammetry under an oxygen atmosphere) [45]. Regardless of the size of the carbon spheres (CNS or CMS), the enclosed area increases with the increase in EG doping. The enhancement in capacitance resulting from an increase in EG content can be attributed to the enhancement of conductivity and graphitization degree in the samples. It is widely recognized that improved electrical conductivity leads to significant enhancements in energy and power densities, as well as specific capacitance [46]. However, it is important to highlight that N-doping has a different effect on nanospheres (CNS) compared to macrospheres (CMS). In both cases, the surface area decreases upon N-doping, but whereas in CMS-5 the capacitance increases with N-doping in CNS-5, the capacitance decreases. Notably, pseudofaradaic peaks are observed in N-CMS-5 but are not clearly identified in N-CNS-5. This can be ascribed to the porosity blockage and different distribution of the nitrogen functional groups. Peng Zhao et al. [47] observed that the capacitance of N-doped NCTs is improved when abundant pyrrolic-N are presented on the surface of the material. K. Tian et al. [48] also identified pyrrolic nitrogen species as highly active pseudocapacitive sites in nitrogen-doped carbon materials employed as supercapacitors. The pseudocapacitance of these carbon materials exhibits a positive correlation with the pyrrolic nitrogen content. In CMS-5, the S_BET_ decreases from 555 to 260 m^2^ g^−1^ upon N-doping, but a high amount of pyrrolitic/pyridonic-N is fixed (around 28%), which compensates for the decrease in porosity and increases the capacitance. In CNS-5, the porosity is highly blocked and fewer highly active pyrrolitic/pyridonic-N (24%) active sites are obtained, which enhances the decrease in pseudocapacity as a result of the porosity blockage.

**Figure 7:**
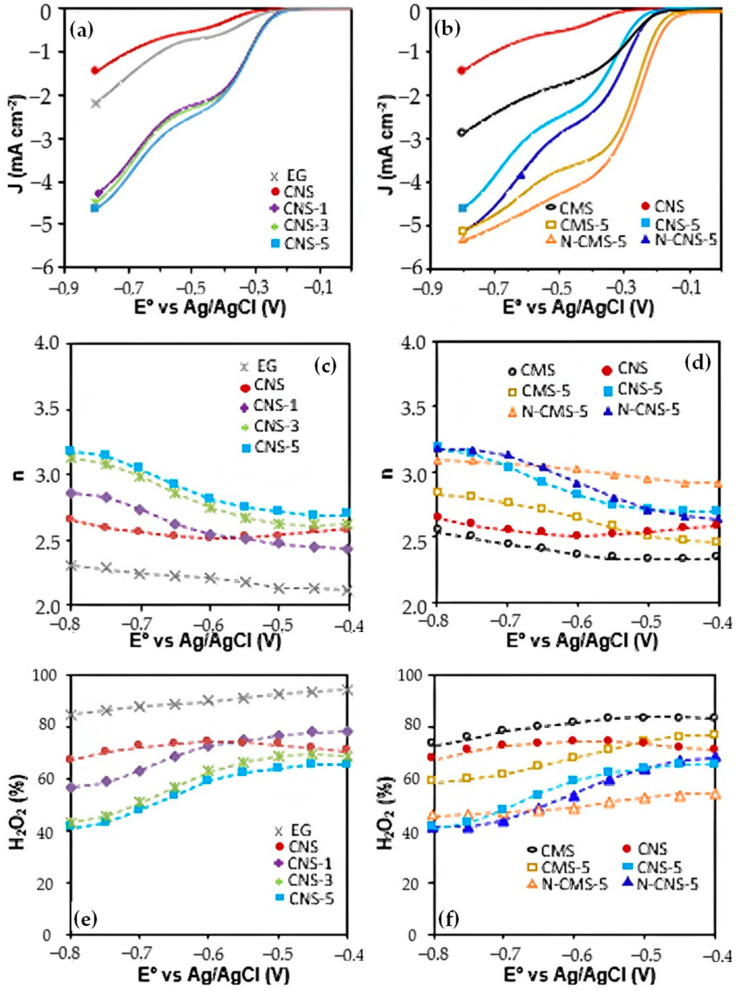
(**a**,**b**) LSV at 3500 rpm, (**c**,**d**) number of electrons transferred and (**e**,**f**) % H_2_O_2_ produced; (**a**,**c**,**e**) reflects the effect of EG doping and (**b**,**d**,**f**) the effect of sphere size and N-doping.

Linear Sweep Voltammetries (LSV) were performed at different rotation speeds to obtain information about the kinetic current density (J_k_) in the ORR as well as the reaction mechanism by determining the number of electrons transferred (n). Figure 7a,b show LSV at 3500 rpm for all the tested materials. The introduction of eco-graphene, both in micro and nanospheres, enhances catalytic activity. As the percentage of eco-graphene increases, greater activity is observed (see J_k_ values in Table 4). This can be attributed to the improved electron transfer to carbon microspheres that is facilitated by eco-graphene, leading to enhanced efficiency in the ORR process. However, note that the activity of carbon microspheres (CMS) is greater that of the carbon nanospheres (CNS) which can be explained based on the intrinsic carbon xerogels defects and the exposed surface area. The intrinsic defects serve as active sites to rapidly adsorb O_2_ and accelerate its transformation [49]. As was confirmed by N_2_-isotherms, the CNS porosity is composed mainly of narrower ultramicropores that are less accessible to the electrolyte and reactants than CMS porosity; thus, CNS has a less active site surface for ORR than CMS. Moreover, Raman spectroscopy shows that the CNS surface presents fewer defects than the CMS surface, providing a smaller number of actives sites for ORR conversion. Figure 7c and d show the number of electron transfers versus the potential for all samples. An almost-pure two-electron pathway was obtained with EG of H_2_O_2_ being the main product (>90%). The two-e^−^ pathway is also the main route for pure microspheres and nanospheres (CMS and CNS) in all potential range. However, the addition of EG does not have a significant effect on the electrons transferred at a potential lower than −0.5, which is (n) in all cases near to 2.5–2.6 (Table 4), similar to the findings for pure carbon spheres. Nonetheless, at a potential higher than −0.5, the addition of EG increases the number of electrons transferred (n), mainly obtaining a 3e^−^ pathway. 

In the electro-Fenton context, the conventional understanding suggests that a two-electron transfer is the typical route for generating hydrogen peroxide in situ (Equation (1)), followed by its conversion into hydroxyl radicals by a Fenton catalyst (Equation (2)). However, previous studies have shown the viability of a three-electron pathway [50] which directly generates ·OH radicals. Recently, Miao et al. [51] proposed a mechanism to elucidate this alternate pathway. According to their proposal, oxygen undergoes reduction to form adsorbed H_2_O_2_, which then directly generates hydroxyl radicals without necessitating desorption (Equation (6)) via one-electron ORR. Thus, the three-electron pathway observed in our samples could be explained based on (i) the materials under consideration potentially possessing active sites capable of facilitating both the reaction outlined in Equation (1), generating H_2_O_2_ via 2e^−^ ORR, and that described in Equation (6) at potential higher than −0.5 V, obtaining ·OH radicals via 1e^−^ ORR, and/or (ii) at this potential, the ORR via 4 electron pathway becoming important and obtaining a competitive production of H_2_O_2_ and H_2_O. L. Xie et al. [52] identified the 3e^−^ pathway in Cu/CoSe_2_/C catalyst, which exhibits remarkable activity for ·OH generation. Using DFT, they performed calculations which demonstrated that Co sites exhibit an easy H_2_O_2_ formation process, but a considerable energy barrier of 0.26 eV is required to form ·OH, meaning that it is favorable for H_2_O_2_ desorption. The transfer of n is almost constant with the voltage for all two-electron samples (EG, CMS, CNS), whereas a mechanism change is observed at −0.5 V for EG-doped samples. This could manifest that the introduction of new sites for H_2_O_2_ conversion to an ·OH radical is the most plausible route in EG-doped samples, rather than the competitive production of water, but potential higher than −0.5 V is required to activate this transformation.

On the other hand, N-doping increases the ORR activity in both CMS and CNS series (Figure 7b and Table 4). It is well known that nitrogen functional groups have been proposed as active sites for the ORR, improving its catalytic performance [29,53]. It is important to highlight that although the N-content fixed on carbon microspheres is double that on carbon nanospheres, the catalytic improvement achieved in nanospheres by N-doping, N-CNS-5, is much higher than in carbon microspheres, N-CMS-5, in relation to their non-N-doped counterpart (see Table 4, J_k_ values). Moreover, the effect of N-doping on the ORR selectivity is very different depending on the size of the carbon sphere (Figure 7d and Table 4). The N doping has a different effect on the selectivity depending on the size of the carbon spheres. The n transferred and its profile vs. E^0^ do not change after N-doping of carbon nanospheres (CNS-5 vs. N-CNS-5), whereas the number of electrons increases to a value higher than three and it is univariable with the potential for N-CMS-5 sample regarding CMS-5. This different behavior is attributed to the different N functional groups’ distribution obtained on the carbon surface depending on the size of the sphere. XPS results revealed that pyridinic and pyrrolitic/pyridonic are the main N-functional groups on carbon microspheres surface, whereas Graphitic-N are predominant in carbon nanospheres. The literature indicated that N-pyridinic tends to enhance the four-electron oxygen reduction reaction (ORR) by facilitating electron donation, whereas N-pyrrolic and graphitic-N, mainly the latter, aid in accelerating the two-electron ORR [24,27,28]. Note that the amount of 2e^−^ ORR actives sites account for 60.5% in N-CNS-5, with 35.7% being graphitic-N groups in comparison with the 43.4% of 2e^−^ ORR active sites and 15.3% of graphitic-N of N-CMS-5. Since mainly N-pyridinic groups are introduced in carbon microspheres, the selectivity to the H_2_O production is enhanced, increasing the n transferred. However, N graphitic dominates in carbon nanospheres and thus, the activity of N-doped CNS increases (see J_k_) but the selectivity to the production of H_2_O_2_ and ·OH radicals remains high. It is important to highlight that N-doping in CMS enhanced the four-electron pathway in all potential ranges, which corroborates the notion that the three-electron pathway observed in EG-doped carbon spheres seems to be ascribed to the production of H_2_O_2_ and its conversion to OH radicals, rather that the enhancement of the four-electron pathway only occurs in a defined range of potentials.

To corroborate the above finding, the H_2_O_2_ generation was determined using a rotating disk electrode (Figure 7e,f). EG, CMS and CNS, which are predominantly 2e^−^ catalysts, produce > 75% of H_2_O_2_ in all potential ranges. Doping with EG favors the introduction of new sites for the direct conversion of H_2_O_2_ to ·OH radicals active at potential higher than −0.5 V, and thus, the amount of H_2_O_2_ detected is around 70% for a potential lower than −0.5 V and then decreases to 40% at higher potential, although the n transferred only increases from 2.7 to 3.1 eV, corroborating the H_2_O_2_ transformation to ·OH radicals.

#### 2.3.2. Size Effect of N-CS in the Catalytic Activity

**Figure 8:**
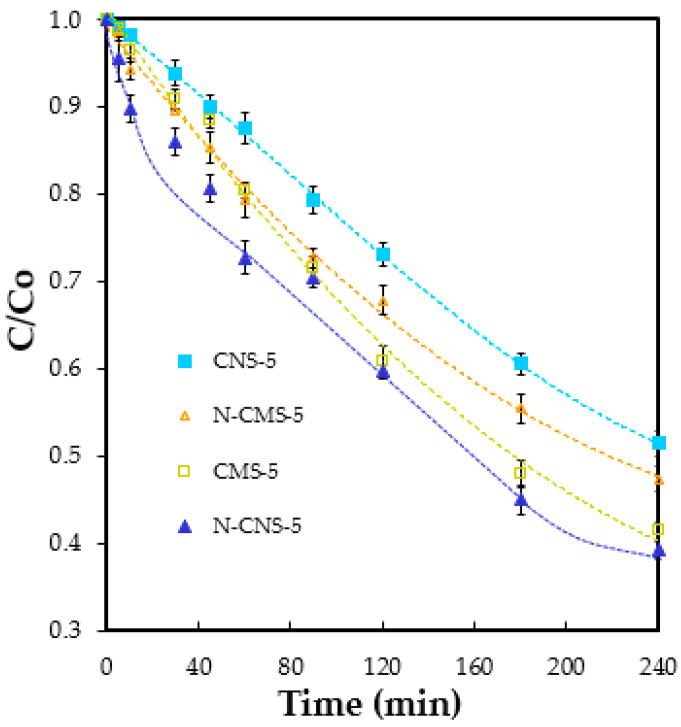
Normalized TTC concentration vs. time at potential of −0.8 V for eco-graphene-doped and N-eco-graphene-doped microspheres and nanospheres.

Since ·OH radicals seem to be produced in EG-CNS and mainly in N-CNS samples, the N-CNS-5 sample is presented as an excellent EF catalyst. Thus, samples doped with nitrogen and 5% of eco-graphene with both carbon spheres sizes (CMS-5, CNS-5, N-CMS-5 and N-CMS-5) were selected to evaluate the catalytic performance in the EF degradation of tetracycline (TTC) at a potential of −0.8 V to ensure high activity and selectivity to OH radicals. To clearly identify the degree of degradation attributed to the electro-Fenton process, the adsorption process was first eliminated by saturating the working electrode with TTC at room temperature, in the dark, and setting the initial concentration at 3.4 × 10^−5^ M. The reaction was then initiated by applying the selected potential with constant O_2_ bubbling, and TTC degradation was monitored over time. The results are shown in Figure 8. As expected, all samples are active in the EF process, which is attributed to the capability of samples to produce ·OH radicals through the electro-reduction of O_2_ via three electrons. It is crucial to emphasize that in a previous study, the possibility of TTC degradation solely through oxidation with H_2_O_2_ or via a simple oxidation–reduction process induced by the applied current was ruled out [34]. However, the EF activity of samples depends on the N-doping and the size of the carbon sphere. A degradation of 48.3% is obtained after 240 min using CNS-5 as an electrode. The activity of their micro-sized counterpart, CMS-5, is 58.6% at 240 min. This higher activity is explained based on the higher ORR activity and thus, OH radicals generation. CMS samples present a more accessible porosity and more defective surface, providing a greater number of actives sites for ORR. When analyzing the effect of the N-doping, it is important to highlight that the introduction of N has a positive or negative effect depending on the size of the carbon spheres, since as it was previously pointed out, the carbon size affects the nature of functional groups anchored on the carbon spheres and, consequently, the ORR selectivity and ·OH radicals production. In this way, while doping with N increases degradation from 48.3% to 61.6% in sample CNS-5, it decreases from 58.6% to 53.4% in sample CMS-5. As was pointed out in ORR, the number of electrons remains invariable after N-doping in the CNS-5 sample, showing a high selectivity to the 3e^−^ pathway, but the activity (see J_k_ values) highly increases due to the selective anchoring of graphitic-N, which are active sites for H_2_O_2_ generation. However, for the CMS-5 sample, the activity increases due to the introduction of active sites for ORR (N-functional groups) but selectivity to ·OH radicals generation decreases due to the enhanced anchoring of pyridinic-N, which are highly selective to H_2_O production.

Considering the obtained results, the miniaturization of the carbon spheres from micro- to nanospheres via more ecofriendly synthesis routes (hydrothermal methods) is an excellent strategy to control the nature of nitrogen functional groups and, thus, enhance the ·OH radicals generation for drug degradation. However, this synthesis method provides carbon nanospheres with a less defective surface and less accessible porosity that do not favor the ORR activity. Thus, in future works, the use of polymerization catalysts could be used to control the carbon sphere size and the pore size distribution, as well as activation protocols to make the designed porosity more accessible and create surface defects to enhance the ORR activity and, thus, the TTC degradation.

In general, our results demonstrate that it is possible to obtain metal-free catalysts capable of directly generating ·OH radicals, making them excellent candidates for new bifunctional electro-Fenton catalysts. Table 5 provides a summary of other bifunctional metal-free catalysts based on carbon materials.

Based on the findings presented in the literature, it is evident that the samples synthesized in this study show promise. Unlike other approaches, our synthesis methods are more environmentally friendly, as they do not involve the use of sulfuric acid. Additionally, operationally, our methods are simpler in some cases. Furthermore, our carbon xerogels exhibit a selectivity closer to three electrons in the ORR compared to those reported in other studies. While the ORR 3e^−^ route is proposed as the principal pathway for the degradation of pollutants by electro-Fenton in these studies, the selectivity values are below 2.6.

## 3. Conclusions

Based on the findings of this study, it can be concluded that the hydrothermal method not only offers operational simplicity but also enables the synthesis of metal-free carbonaceous catalysts with bifunctional activity in electro-Fenton processes for tetracycline degradation. Furthermore, doping with EG significantly boosts the current density of the electrocatalytic reaction, while parameter n tends to increase. 

The size of the carbon spheres was identified as a critical factor in the impact of nitrogen doping. For instance, in CNS-5, the degradation of TTC increased from 48.3% to 61.6%, whereas in CMS-5 it decreased from 58.6% to 53.4%. This disparity can be primarily attributed to the nitrogenous groups present in each sample, as revealed by XPS analysis. Specifically, the N-CMS-5 sample exhibited a higher concentration of pyridinic nitrogen, which favors water production, whereas the N-CNS-5 sample had a higher concentration of graphitic nitrogen, which promotes H_2_O_2_ generation. 

## 4. Materials and Methods

### 4.1. Synthesis of Materials

#### 4.1.1. Eco-Graphene (EG)

To achieve a more environmentally friendly process, the synthesis of eco-graphene (EG) was conducted according to procedures reported in the literature [57]. The process involved hydrolyzing glucose in ammonia/CTAB solution followed by a hydrothermal process. CTAB was dissolved in a 0.5 M glucose solution with a CTAB/glucose molar ratio of 1.5/8. Ammonium hydroxide was then added to adjust the pH to 11. The solution was treated in an autoclave at 270 °C for 4 h, cooled slowly, and the product was collected by filtration. After being washed with distilled water, it was dried at 70 °C under vacuum for two days.

#### 4.1.2. Carbon Xerogel Spheres (CS)

We synthesized CS using two different methods, aiming to employ an operationally simpler process (solvothermal) compared to one that offers more favorable conditions for synthesis (65 °C in atmospheric pressure).

The carbon xerogel nanospheres (CNS) were synthesized with different percentages of EG (0, 1, 3, and 5 wt.%). The synthesis was carried out following a typical solvothermal process [58]. Briefly, a solution composed of 67.2 mL of ethanol (98%), 168 mL of deionized water, and 1.5 mL of NH_4_OH (28%) was prepared. Then, 1.68 g of resorcinol was dissolved and the appropriate quantity of eco-graphene for each doping percentage was dispersed. Finally, 2.35 mL of formaldehyde was added, and the mixture was sonicated for 10 min. The homogeneous solution was transferred to a Teflon reactor in a stainless-steel autoclave and treated at 100 °C for 24 h. The obtained solid was recovered by centrifugation and immersed in acetone. The acetone was changed twice daily for three days to exchange the water within the pores, with the aim of reducing the porosity shrinkage during the subsequent drying process. Finally, the organic gel was dried using a microwave oven (800 W) under argon flow at 300 W during a 1 min cycle until it reached a constant weight and then carbonized at 850 °C for 2 h with a heating rate of 1.5 °C min^−1^ under a continuous flow of nitrogen gas (150 mL min^−1^). 

The carbon xerogels spheres with micrometric size (CMS) were obtained by an inverse microemulsion polymerization of resorcinol (R) and formaldehyde (F) within an organic medium [35]. To achieve this, a mixture composed of R, F and water (W) in the molar ratios of R/F = 0.5 and R/W = 0.067 was prepared. Then, the corresponding amount of EG required to obtain the desired wt % in the final carbon material was added and the mixture was sonicated for 10 min. The mixture was pre-gelled for 1 h at 65 °C in a sealed vessel and then added dropwise to a solution of 22 mL of Span 80 (S) and 900 mL of n-heptane under stirring (650 rpm) and reflux at 65 °C. The molar ratio of R/S was 4.42. This solution was maintained at 65 °C under reflux and stirring for 24 h. Subsequently, the gel was filtered and immersed in acetone. The acetone was replaced twice daily for five days to aid in the exchange of water within the pores and removal of the surfactant Span 80. Finally, the organic gels were dried and carbonized in the same conditions described above.

The samples were labeled as carbon xerogels micro or nanospheres (CMS of CNS, respectively) followed by the wt % of EG in the final carbon xerogel, e.g., CMS-3 means that carbon xerogel microspheres were doped with 3 wt.% of eco-graphene.

#### 4.1.3. N-Doped Carbon Xerogel Spheres (N-CS)

The N doping of carbon spheres was carried out using melamine as nitrogen source. The melamine in a mass melamine/carbon of 1:1 was dissolved in ethanol (98%) and mixed with the carbon xerogel. The mixture was stirred for 2 h and then dried in infrared light. Finally, the solid was thermically treated at 650 °C for 1 h under a N_2_ flow (150 mL min^−1^) with a heating rate of 10 °C min^−1^. The samples were labeled as N-CYS-Z, where Y is the sphere size (M: Micro or N: nano), Z is the EG wt % and N indicates the nitrogen doping (if there is no “Y”, it means that N-doping was not performed).

### 4.2. Characterization

#### 4.2.1. Chemical and Textural Characterization

The textural properties of samples were studied by N_2_ adsorption isotherms at 77 K. the Brunauer–Emmett–Teller (B.E.T) method, Dubinin–Radushkevic (DR) equation, and density functional theory (DFT) were applied to obtain the specific surface area (S_BET_), micropore volume (W_0_) and width (L_0_), and the pore size distribution, respectively. The N_2_ volume adsorbed at the relative pressure of 0.95 was used as the total pore volume (V_0.95_). The mesopore volume (V_meso_) was calculated as the difference between V_0.95_ and W_0_.

Raman spectroscopy was used to analyze the graphitization degree of the samples. The spectra were reordered at 24 Mw in a range from 200 to 3000 cm^−1^ using a Micro-Raman JASCO NRS-5100 dispersive spectrophotometer equipped with a 532 nm laser. 

The surface composition was determined by X-ray photoelectron spectroscopy (XPS) using a Kratos Axis Ultra-DLD spectrometer equipped with a hemispherical electron analyzer connected to a detector DLD (delay-line detector) and an Al-Kα monochromator of a power of 600 W. The C_1s_ peak position at 284.6 eV was used as internal reference for binding energy correction.

The morphology of the samples was studied by scanning electron microscopy (SEM) in an AURIGA FIB-FESEM microscope and by transmission electron microscopy (TEM) in a LIBRA 120 Plus microscope. Microphotographs were analyzed by the appropriate software (ImageJ 1.54f) to obtain the spheres size distributions. 

#### 4.2.2. Electrochemical Characterization

The electrochemical characterization was performed in a Biological VMP Multichannel potentiostat using a rotating ring-disk electrode (RRDE) as working electrode, a Ag/AgCl as a reference electrode, and a Pt wire as a counter electrode. The carbon spheres-based samples were deposited onto the Glassy Carbon tip of the RRDE. To achieve this, an ink composed of 5 mg of sample and 1 mL of Nafion water solution in a volumetric ratio of 1:9 (Nafion 5% solution water) was prepared, and 20 mL of this ink was deposited on the tip and was dried by infrared radiation.

Cyclic voltammetries (CVs) were conducted in N_2_- or O_2_-saturated 0.1 M KOH solutions in a range from 0.4 V to −0.8 V (vs. Ag/AgCl) at two scan rates (5 mV s^−1^ and 50 mV s^−1^) while the RRDE rotated at 1000 rpm. The linear sweep voltammetry (LSV) was carried out in O_2_-saturated 0.1 M KOH solutions at different rotation rates (500, 1000, 1500, 2000, 2500, 3000, 3500, 4000 rpm) in a working window from 0.4 to −0.8 V (vs. Ag/AgCl) at a sweep rate of 5 mV s^−1^. The LSV was fitted to the Koutecky–Levich model to calculate the number of electrons transferred (n) and the kinetic density current (J_k_). Based on the disk and platinum ring current measurements, the overall electron transfer number (n) and the H_2_O_2_% were calculated by means of Equations (7) and (8), respectively.

(7) $$n=\frac{4\cdot I_{D}}{I_{D}-\frac{I_{R}}{N}}$$

(8) $$H_{2}O_{2}\left(\%\right)=100\cdot \frac{2\cdot \frac{I_{R}}{N}}{I_{D}-\frac{I_{R}}{N}}$$

where I_R_ and I_D_ are the ring and disk currents, respectively, and N is the collection efficiency of RRDE (0.245).

### 4.3. Electro-Fenton Processes

The electro-Fenton process was carried out in a three-electrode glass cell controlled by a Biological VMP multichannel potentiostat (BioLogic Science Instruments VMP3 0216, Granada) using Ag/AgCl as reference electrode, a Pt-wire as the counter electrode and graphite paper on which the sample was pasted as the EF working electrode. For the preparation of the EF electrodes, a homogeneous paste was prepared by mixing finely milled carbon xerogel spheres and polytetrafluoroethylene (PTFE) binder in a mass ratio of 9:1. The paste was dried at 80 °C overnight and, finally, 50 mg of this paste was coated on graphite paper with an area of 3 cm × 1 cm. 

To study the dual-functional electrocatalysts in electro-Fenton (EF) tests, tetracycline (TTC) was chosen as the emerging pollutant. Tetracycline adsorption kinetics and isotherms were performed on the working electrode prior to the EF tests to determine the amount of time required to achieve the adsorption equilibrium and the adsorption capacity, respectively. Once the adsorption capacity had been determined, the working electrode was put in contact with a tetracycline solution of the appropriate TTC concentration in 0.5 M Na_2_SO_4_ to obtain a final concentration of 3.4 × 10^−5^ M after reaching the adsorption equilibrium. Once the adsorption equilibrium was achieved, the TTC solution was saturated with bubbling O_2_ for 30 min before the EF experiment started and continuously bubbled throughout the experiment. During the EF experiment, 1 mL aliquots were periodically taken from the glass cell at the specified time intervals. The concentration of TTC in each aliquot was immediately analyzed at 358 nm using a UV-spectrophotometer model UV-2600i Shimadzu.

## Figures and Tables

**Table 1:** Textural characteristics of all samples obtained by N_2_ adsorption at 77K.

Sample	N_2_-Isotherm					CO_2_ Isotherm		Raman		
	S_BET_	W_0_	L_0_	V_0.95_	V_meso_	W_0_	L_0_	I_D_/I_G_	D Position	G Position
	m^2^g^−1^	cm^3^g^−1^	nm	cm^3^g^−1^	cm^3^g^−1^	cm^3^g^−1^	nm	n.a.	cm^−1^	cm^−1^
EG	1	0.00	-	0.00	0.00	-	-	0.78	1363	1563
CNS	560	0.23	0.51	0.27	0.00			1.00	1344	1588
CNS-1	-	-	-	-	-	-	-	0.98	1340	1587
CNS-3	-	-	-	-	-	-	-	0.98	1341	1587
CNS-5								0.97	1341	1585
N-CNS-5								0.98	1338	1583
CMS	501	0.20	1.19	0.23	0.03	0.26	0.56	1.05	1343	1590
CMS-5	555	0.23	0.86	0.41	0.18	0.24	0.56	1.00	1340	1587
N-CMS-5	260	0.11	1.60	0.32	0.21	0.24	0.56	1.01	1343	1590

**Table 2:** Analysis of the elemental composition.

Sample	Elemental Composition (wt.%)			
	C	H	O	N
CNS	95.46	0.34	4.15	0.05
N-CNS	87.15	0.95	8.67	3.23
CNS-1	95.38	0.33	4.20	0.09
CNS-3	95.00	0.33	4.53	0.14
CNS-5	95.93	0.35	3.51	0.21
N-CNS-5	89.18	0.67	7.74	2.41
CMS	95.96	0.33	3.71	0.00
CMS-5	96.00	0.35	3.42	0.23
N-CMS-5	90.10	0.25	3.57	6.08

**Table 3:** Surface chemical composition determined by XPS.

Sample	C_1s_			O_1s_			N_1s_			C_XPS_ (%)	O_XPS_ (%)	N_XPS_ (%)
	BE (eV)	% Peak	Assign.	BE (eV)	% Peak	Assign.	BE (eV)	% Peak	Assign.			
CMS	284.6	59.3	C=C	532.4	54.1	C=O				96.1	3.9	-
	285.3	23.1	C–C	533.8	45.9	C-O						
	286.6	8.0	C–O									
	288.1	3.3	C=O									
	289.8	4.1	COO−									
	291.4	2.2	π−π*									
N-CMS-5	284.6	55.2	C=C	530.8	12.2	Quin.	398.4	56.6	Pyridinic	92.4	2.5	5.1
	285.3	23.4	C–C	532.3	50.3	C=O	399.8	28.1	Pyrrolitic/Pyridonic			
	286.6	9.7	C–O	533.5	37.5	C-O	401.0	15.3	Graphitic			
	288.0	4.9	C=O									
	289.9	4.2	COO−									
	291.5	2.6	π−π*									
CNS	284.6	56.6	C=C	530.7	8.1	Quin.	398.4	23.7	Pyridinic	92.3	7.1	0.6
	285.3	23.7	C–C	532.3	53.2	C=O	399.9	11.1	Pyrrolitic/Pyridonic			
	286.6	9.4	C–O	533.6	38.7	C-O	401.0	65.2	Graphitic			
	288.0	4.2	C=O									
	289.3	3.9	COO−									
	290.9	2.2	π−π*									
CNS-5	284.6	55.8	C=C	530.7	6.2	Quin.	398.4	47.4	Pyridinic	90.9	8.7	0.4
	285.4	23.1	C–C	532.3	50.1	C=O	399.9	22.7	Pyrrolitic/Pyridonic			
	286.6	10.9	C–O	533.7	43.7	C-O	400.9	29.9	Graphitic			
	288.0	4.4	C=O									
	289.3	3.9	COO−									
	290.9	1.9	π−π*									
N-CNS-5	284.6	57.8	C=C	530.8	13.8	Quin.	398.3	39.5	Pyridinic	85.6	5.2	9.1
	285.4	22.2	C–C	532.3	59.3	C=O	399.8	24.8	Pyrrolitic/Pyridonic			
	286.6	8.9	C–O	533.5	26.8	C-O	401.0	35.7	Graphitic			
	288.2	5.2	C=O									
	289.8	3.4	COO−									
	291.5	2.5	π−π*									
Eco-G	284.5	72.8	C=C	530.6	30.9	Quinone	398.4	47.4	Pyridinic	75.7	11.3	13.1
	285.7	20.9	C–C	532.0	69.1	C=O	399.6	47.2	Pyrrolitic/Pyridonic			
	286.9	6.3	C–O				401.8	5.4	Graphitic			

**Table 4:** Electrochemical parameters obtained from the analysis of LSV curves.

Sample	E_onset_ (V)	J_k_ mAcm^−2^		n		H_2_O_2_ (%)	
		−0.8 V	−0.4 V	−0.8 V	−0.4 V	−0.8 V	−0.4 V
EG	−0.26	5.27	0.67	2.31	2.12	84.72	94.10
CNS	−0.28	1.90	0.28	2.65	2.58	67.36	70.76
CNS-1	−0.23	9.62	2.89	2.87	2.54	56.63	78.24
CNS-3	−0.23	10.05	2.95	3.13	2.63	43.28	68.60
CNS-5	−0.23	10.81	3.07	3.18	2.69	40.99	65.27
N-CNS-5	−0.22	14.13	3.96	3.18	2.64	41.10	68.05
CMS	−0.19	4.60	1.99	2.54	2.34	73.04	83.18
CMS-5	−0.18	14.16	8.66	2.83	2.47	58.47	76.72
N-CMS-5	−0.15	16.10	9.45	3.10	2.91	45.62	54.43

**Table 5:** Bifunctional catalysts employed in the electro Fenton degradation process, as reported in the literature.

Catalyst	n	Pollutant	Experiment Conditions	Time (min)	%Degradation	Ref
OCNT-80 (O-doped carbon nanotubes)	2.5 to 2.6 (−0.3 to −1.0V vs. SCE)	Phenol	−0.4V vs. SCE, pH = 6.5	60	99.2	[49]
NGE (Nitrogen-doped Graphene)	2.1 to 2.5 (−0.6 to −1.2V vs. Ag/AgCl)	Phenol	pH neutral	180	93.6	[54]
PPC (O and F doped porous carbon)	2.1 to 2.2 (−0.4 to −1.6V vs. Ag/AgCl)	Sulfamerazine	−1.5V vs. Ag/AgCl, pH = 3	180	90.1	[55]
ACSS (Activated carbon wrapped with stainless steel)	-----	RB19	100 mA, pH = 7	720	61.5	[12]
N,S-EEGr (Nitrogen and sulfur co-doped graphene)	2.22 to 2.27 (−1.0 to 0.0V vs. SCE)	Phenol	6.25 mA cm^−2^, pH = 7	15	100	[56]

## Data Availability

The data presented in this study are openly available in the article.

## References

[1] Song Y., Meng C., Lyu Y., Liu Y., Li Y., Jiang Z., Jiang K., Hu C. (2024). Self-Cleaning Foulant Attachment on near-Infrared Responsive Photocatalytic Membrane for Continuous Dynamic Removing Antibiotics in Sewage Effluent Environment. Water Res..

[2] Fang Y., Lin G., Liu Y., Zhang J. (2024). Advanced Treatment of Antibiotic-Polluted Wastewater by a Consortium Composed of Bacteria and Mixed Cyanobacteria. Environ. Pollut..

[3] Chen A., Wang H., Zhan X., Gong K., Xie W., Liang W. (2024). Applications and Synergistic Degradation Mechanisms of NZVI-Modified Biochar for the Remediation of Organic Polluted Soil and Water: A Review. Sci. Total Environ..

[4] Li S., Gao M., Dong H., Jiang Y., Liang W., Jiang J., Ho S.H., Li F. (2022). Deciphering the Fate of Antibiotic Resistance Genes in Norfloxacin Wastewater Treated by a Bio-Electro-Fenton System. Bioresour. Technol..

[5] Zhong D., Zhang J., Huang J., Ma W., Li K., Li J., Zhang S., Li Z. (2023). Accelerated Electron Transfer Process via MOF-Derived FeCo/C for Enhanced Degradation of Antibiotic Contaminants towards Heterogeneous Electro-Fenton System. Chemosphere.

[6] Hu J., Cheng W., Zhao Y., Song Y., Xu H., Zhou H., Yang H., Sun J., Chi R. (2024). The Synergistic Effect of Oxygen Vacancies and Acidic Properties of SO42−/CexZr1−xO2 on Enhancing the Electro-Fenton Performance in Antibiotics Wastewater Treatment. Sep. Purif. Technol..

[7] Xu A., Liu W., Yang Z., Cao L., Sirés I., Zhang Q., Zhang Y. (2023). Waste Tire Upcycling for the Efficient Electrogeneration of H2O2 in Advanced Degradation of the Antibiotic Tinidazole by Electro-Fenton Process. J. Clean. Prod..

[8] Lu W., Chen N., Feng C., Deng Y., Feng Z., Hu Y., Liu T., Hu W. (2023). A Bifunctional Graphene-Based Cathode for Wastewater Treatment in Heterogeneous Electro-Fenton: Taking Textile, Old Landfill Leachate and Simulated Antibiotic Wastewater as Examples. Chem. Eng. J..

[9] Lai S., Zhao H., Qu Z., Tang Z., Yang X., Jiang P. (2022). Promotion of Formaldehyde Degradation by Electro-Fenton: Controlling the Distribution of ⋅ OH and Formaldehyde near Cathode to Increase the Reaction Probability. Chemosphere.

[10] Li Y., Yao B., Chen Y., Zhou Y., Duan X. (2023). Metal-Organic Frameworks (MOFs) as Efficient Catalysts for Electro-Fenton (EF) Reactions: Current Progress and Prospects. Chem. Eng. J..

[11] Robles I., Martínez R.J., Banda-alem J.A., Salazar-l M.L., Manríquez J., García-espínoza D., Godínez L.A. (2023). Surface Oxidation Pre-Treatment on Activated Carbon: Effect on Its Cathode Performance in Electro-Fenton Processes. Mater. Today Commun..

[12] Zhou W., Rajic L., Chen L., Kou K., Ding Y., Meng X., Wang Y., Mulaw B., Gao J., Qin Y. (2019). Activated Carbon as Effective Cathode Material in Iron-Free Electro-Fenton Process: Integrated H2O2 Electrogeneration, Activation, and Pollutants Adsorption. Electrochim. Acta.

[13] Babaei-sati R., Parsa J.B. (2017). Electrogeneration of H 2 O 2 Using Graphite Cathode Modi Fi Ed with Electrochemically Synthesized Polypyrrole / MWCNT Nanocomposite for Electro-Fenton Process. J. Ind. Eng. Chem..

[14] Rivera-vera C., Rodrigo-rodrigo M.A., Saez C., Thiam A., Salazar-gonz R. (2024). Electrogeneration of H2O2 through Carbon-Based Ink on Al Foam for Electro-Fenton Treatment of Micropollutants in Water. Chemosphere.

[15] Trench A.B., Moura J.P.C., Antonin V.S., Gentil T.C., Lanza M.R.V., Santos M.C. (2023). Using a Novel Gas Diffusion Electrode Based on Vulcan XC-72 Carbon Modified with Nb2O5 Nanorods for Enhancing H2O2 Electrogeneration. J. Electroanal. Chem..

[16] Hu X., Wang J., Jin T., Li Z., Tsang Y.F., Liu B. (2022). Efficient H2O2 Generation and Bisphenol A Degradation in Electro-Fenton of O-Doped Porous Biochar Cathode Derived from Spirit-Based Distiller’s Grains. Process Saf. Environ. Prot..

[17] Wang W., Li W., Li H., Xu C., Zhao G., Ren Y. (2022). Kapok Fiber Derived Biochar as an Efficient Electro-Catalyst for H2O2 in-Situ Generation in an Electro-Fenton System for Sulfamethoxazole Degradation. J. Water Process Eng..

[18] Zhang Y., Zhan L., Hu L., Fan G. (2023). Oxygen-Assisted One-Step Fabrication and Concomitant Regulation of Hierarchical Carbon Nanosheet Assemblies with Conjugated Binding Configurations for Promoted H2O2 Electrosynthesis. Fuel.

[19] Wu F., Nan J., Wang T., Ge Z., Liu B., Chen M., Ye X. (2023). Highly Selective Electrosynthesis of H2O2 by N, O Co-Doped Graphite Nanosheets for Efficient Electro-Fenton Degradation of p-Nitrophenol. J. Hazard. Mater..

[20] Wang X., Liu Y., Liu Z., Li Z., Zhang T., Cheng Y., Lei L., Yang B., Hou Y. (2023). Highly Efficient Electrosynthesis of H2O2 in Acidic Electrolyte on Metal-Free Heteroatoms Co-Doped Carbon Nanosheets and Simultaneously Promoting Fenton Process. Chinese Chem. Lett..

[21] Wang Y., Waterhouse G.I.N., Shang L., Zhang T. (2021). Electrocatalytic Oxygen Reduction to Hydrogen Peroxide: From Homogeneous to Heterogeneous Electrocatalysis. Adv. Energy Mater..

[22] Yang Y., He F., Shen Y., Chen X., Mei H., Liu S., Zhang Y. (2017). A Biomass Derived N/C-Catalyst for the Electrochemical Production of Hydrogen Peroxide. Chem. Commun..

[23] Han S., Wang Z., Pi X., Wu C., Wang X., Wang Y., Liu X., Zhao H. (2022). Promotion of Tetracycline Degradation by Electro-Fenton: Controlling the Reaction Zone by N-Doped Modified Activated Carbon Cathode. J. Clean. Prod..

[24] Zhu Y., Qiu S., Deng F., Ma F., Zheng Y. (2020). Degradation of Sulfathiazole by Electro-Fenton Using a Nitrogen-Doped Cathode and a BDD Anode: Insight into the H2O2 Generation and Radical Oxidation. Sci. Total Environ..

[25] Sun Y., Li S., Jovanov Z.P., Bernsmeier D., Wang H., Paul B., Wang X., Kühl S., Strasser P. (2018). Structure, Activity, and Faradaic Efficiency of Nitrogen-Doped Porous Carbon Catalysts for Direct Electrochemical Hydrogen Peroxide Production. ChemSusChem.

[26] Zhang J., Zhang G., Jin S., Zhou Y., Ji Q., Lan H. (2020). Graphitic N in Nitrogen-Doped Carbon Promotes Hydrogen Peroxide Synthesis from Electrocatalytic Oxygen Reduction. Carbon N. Y..

[27] Tian M., Zhu Y., Zhang D., Wang M., Chen Y., Yang Y., Gao S. (2019). Pyrrolic-Nitrogen-Rich Biomass-Derived Catalyst for Sustainable Degradation of Organic Pollutant via a Self-Powered Electro-Fenton Process. Nano Energy.

[28] Chen J., Wang X., Cui X., Yang G., Zheng W. Amorphous Carbon Enriched with Pyridinic Nitrogen as an Efficient Metal-Free Electrocatalyst for Oxygen Reduction Reaction. 2014, 557–559.

[29] Quílez-Bermejo J., Melle-Franco M., San-Fabián E., Morallón E., Cazorla-Amorós D. (2019). Towards Understanding the Active Sites for the ORR in N-Doped Carbon Materials through Fine-Tuning of Nitrogen Functionalities: An Experimental and Computational Approach. J. Mater. Chem. A.

[30] Zhe-lun P., Xu-fang Q. (2022). Porous Carbons for Use in Electro-Fenton and Fenton-like Reactions. NEW CARBON Mater..

[31] Tan Z., Qin X., Cao P., Chen S., Yu H., Su Y., Quan X. (2023). Enhanced Electrochemical-Activation of H2O2 to Produce •OH by Regulating the Adsorption of H2O2 on Nitrogen-Doped Porous Carbon for Organic Pollutants Removal. J. Hazard. Mater..

[32] Tan L., Liu Y., Zhu G., Fan X., Quan X. (2023). Metal-Free Electro-Fenton Degradation of Perfluorooctanoic Acid with Efficient Ordered Mesoporous Carbon Catalyst. Sci. Total Environ..

[33] Yang Q., Chu L., Wu T., Zhou Y., Liu C., Cang L. (2023). Investigation of Dual-Functional Carbon Cathode Catalysts from Agricultural Wastes in the Heterogeneous Electro-Fenton Process. Appl. Catal. B Environ..

[34] Barranco Lopez A., Morales-Rodríguez A.I., Fajardo-Puerto E., Elmouwahidi A., Bailón-García E. (2023). Highly Graphitic Fe-Doped Carbon Xerogels as Dual-Functional Electro-Fenton Catalysts for the Degradation of Tetracycline in Wastewater. Environ. Res..

[35] Ramírez-Valencia L.D., Bailón-García E., Moral-Rodríguez A.I., Carrasco-Marín F., Pérez-Cadenas A.F. (2023). Carbon Gels–Green Graphene Composites as Metal-Free Bifunctional Electro-Fenton Catalysts. Gels.

[36] Alegre C., Sebastián D., Lázaro M.J. (2019). Carbon Xerogels Electrochemical Oxidation and Correlation with Their Physico-Chemical Properties. Carbon N. Y..

[37] Zhao M., Ma X., Yan S., Xiao H., Li Y., Hu T., Zheng Z., Jia J., Wu H. (2020). Solvothermal Synthesis of Oxygen-Incorporated MoS2-x Nanosheets with Abundant Undercoordinated Mo for Efficient Hydrogen Evolution. Int. J. Hydrogen Energy.

[38] Dibale A.A., Su W.-N., Tamirat A.G., Pan C.-J., Aragaw B.A., Chen H.-M., Chen C.H., Hwang B.-J. (2014). The Synergetic Effect of Graphene on Cu2O Nanowire Arrays as a Highly Efficient Hydrogen Evolution Photocathode in Water Splitting. Mater. Chem. A.

[39] Wassner M., Eckardt M., Reyer A., Diemant T., Elsaesser M.S., Behm R.J., Hüsing N. Synthesis of Amorphous and Graphitized Porous Nitrogen- Doped Carbon Spheres as Oxygen Reduction Reaction Catalysts. 2020, 1–15.

[40] Hunter R.D., Hayward E.C., Smales G.J., Kulak A., De S.G., Schnepp Z. (2023). The Effect of Nitrogen on the Synthesis of Porous Carbons by Iron-Catalyzed Graphitization. Mater. Adv..

[41] Gong Y., Shi G., Zhang Z., Zhou W., Jung J., Gao W., Ma L., Yang Y., Yang S., You G. (2014). Direct Chemical Conversion of Graphene to Boron- and Nitrogen- and Carbon-Containing Atomic Layers. Nat. Commun..

[42] Elmouwahidi A., Bailón-García E., Pérez-Cadenas A.F., Castelo-Quibén J., Carrasco-Marín F. (2019). Carbon-Vanadium Composites as Non-Precious Catalysts for Electro-Reduction of Oxygen. Carbon N. Y..

[43] Rufford T.E., Hulicova-jurcakova D., Zhu Z., Lu G.Q. (2009). Empirical Analysis of the Contributions of Mesopores and Micropores to the Double-Layer Capacitance of Carbons. J. Phys. Chem..

[44] Vallerot J., Bourrat X., Mouchon A., Chollon G. (2006). Quantitative Structural and Textural Assessment of Laminar Pyrocarbons through Raman Spectroscopy, Electron Diffraction and Few Other Techniques. Carbon N. Y..

[45] Elmouwahidi A., Bailón-garcía E., Pérez-cadenas A.F., Maldonado-hódar F.J., Carrasco-marín F. (2017). Activated Carbons from KOH and H3PO4 Activation of Olive Residues and Its Application as Supercapacitor Electrodes. Electrochim. Acta.

[46] Canal-rodríguez M., Arenillas A., Men J.A., Ramos-fern G., Rodríguez-pastor I., Martin-gullon I. (2018). Determinant in Fluence of the Electrical Conductivity versus Surface Area on the Performance of Graphene Oxide-Doped Carbon Xerogel Supercapacitors. Carbon N. Y..

[47] Zhao P., Shen B., Yang M., Chen L., Shi G., Lu F. (2023). Effect of Nitrogen Species on Electrochemical Properties of N-Doped Carbon Nanotubes Derived from Co-Pyrolysis of Low-Density Polyethylene and Melamine. J. Energy Storage.

[48] Tian K., Wang J., Cao L., Yang W., Guo W., Liu S., Li W., Wang F., Li X., Xu Z. (2020). Single-Site Pyrrolic-Nitrogen-Doped Sp2-Hybridized Carbon Materials and Their Pseudocapacitance. Nat. Commun..

[49] Qin X., Zhao K., Quan X., Cao P., Chen S., Yu H. (2021). Highly Efficient Metal-Free Electro-Fenton Degradation of Organic Contaminants on a Bifunctional Catalyst. J. Hazard. Mater..

[50] Noël J., Latus A., Lagrost C., Volanschi E., Hapiot P. (2012). Evidence for OH Radical Production during Electrocatalysis of Oxygen Reduction on Pt Surfaces: Consequences and Application. Am. Chem. Soc..

[51] Miao F., Gao M., Yu X., Xiao P., Wang M., Wang Y., Wang S., Wang X. (2020). TiO2 Electrocatalysis via Three-Electron Oxygen Reduction for Highly Efficient Generation of Hydroxyl Radicals. Electrochem. commun..

[52] Xie L., Wang P., Zheng W., Zhan S., Xia Y., Liu Y., Yang W., Li Y. (2023). The Strong Metal–Support Interactions Induced Electrocatalytic Three-Electron Oxygen Reduction to Hydroxyl Radicals for Water Treatment. Environ. Sci..

[53] Skorupska M., Ilnicka A., Lukaszewicz J.P. (2021). The Effect of Nitrogen Species on the Catalytic Properties of N-Doped Graphene. Sci. Rep..

[54] Su P., Zhou M., Song G., Du X., Lu X. (2020). Efficient H2O2 Generation and Spontaneous [Rad]OH Conversion for in-Situ Phenol Degradation on Nitrogen-Doped Graphene: Pyrolysis Temperature Regulation and Catalyst Regeneration Mechanism. J. Hazard. Mater..

[55] Li Y., Cao W.X., Zuo X.J. (2022). O- and F-Doped Porous Carbon Bifunctional Catalyst Derived from Polyvinylidene Fluoride for Sulfamerazine Removal in the Metal-Free Electro-Fenton Process. Environ. Res..

[56] Yang W., Zhou M., Mai L., Ou H., Oturan N., Oturan M.A., Zeng E.Y. (2021). Generation of Hydroxyl Radicals by Metal-Free Bifunctional Electrocatalysts for Enhanced Organics Removal. Sci. Total Environ..

[57] Mohamed M.A.A., Elessawy N.A., Carrasco-Marín F., Hamad H.A.F. (2019). A Novel One-Pot Facile Economic Approach for the Mass Synthesis of Exfoliated Multilayered Nitrogen-Doped Graphene-like Nanosheets: New Insights into the Mechanistic Study. Phys. Chem. Chem. Phys..

[58] Moreno-castilla C., García-rosero H., Carrasco-marín F. (2017). Synthesis and Characterization of Solid Polymer and Carbon Spheres Derived from an Emulsion Polymerization Reaction of Different Phenolic Compounds with Formaldehyde. Colloids Surfaces A Physicochem. Eng. Asp..

